# Informal Payments by Patients in Central and Eastern Europe during the COVID-19 Pandemic: An Institutional Perspective

**DOI:** 10.3390/ijerph182010914

**Published:** 2021-10-17

**Authors:** Adrian V. Horodnic, Colin C. Williams, Răzvan Ionuț Drugă, Cristian Incaltarau

**Affiliations:** 1 Faculty of Medicine, “Grigore T. Popa” University of Medicine and Pharmacy, 700115 Iași, Romania; drugarazvanionut@gmail.com (R.I.D.); cristian.incaltarau@uaic.ro (C.I.); 2 Management School, University of Sheffield, Sheffield S10 2TN, UK; c.c.williams@sheffield.ac.uk; 3 Faculty of Economics and Business Administration, “Alexandru Ioan Cuza” University of Iași, 700505 Iași, Romania; 4 Centre for European Studies, “Alexandru Ioan Cuza” University of Iași, 700507 Iași, Romania

**Keywords:** formal institutions, informal institutions, informal payments, public healthcare system, trust, transparency, performance

## Abstract

Confronted with a global pandemic, public healthcare systems are under pressure, making access to healthcare services difficult for patients. This provides fertile ground for using illegal practices such as informal payments to gain access. This paper aims to evaluate the use of informal payments by patients during the COVID-19 pandemic and the institutions that affect the prevalence of this practice. Various measurements of formal and informal institutions are here investigated, namely the acceptability of corruption, the level of trust, transparency, and performance of the healthcare system. To do so, a logistic regression of 10,859 interviews with patients conducted across 11 Central and Eastern Europe countries in October–December 2020 is employed. The finding is that there are large disparities between countries in the prevalence of informal payments, and that the practice is more likely to occur where there are poorer formal and informal institutions, namely higher acceptability of corruption, lower trust in authorities, lower perceived transparency in handling the COVID-19 pandemic, difficult access to, and poor quality of, healthcare services, and higher mortality rates due to the COVID-19 pandemic. These findings suggest that policy measures for tackling informal payments need to address the current state of the institutional environment.

## 1. Introduction

The COVID-19 pandemic represents one of the most important and disruptive events in the health sector for some decades. Due to its rapid spread throughout the world, there has been a growing demand for access to health services. This has caused the growth in some countries of informal payments to gain preferential access to health services [1]. These informal payments are known by many different names including: ‘bribes/bribe payments’; ‘envelope payments’; ‘gratitude payments’; ‘informal payments’; ‘red packages/envelopes’; ‘under-the-table payments’, and ‘unofficial payments/fees’ [2]. In relation to the healthcare sector, these informal payments represent an exchange, in cash or in kind, in which the employees of the medical units and patients are involved, so that the latter benefit from certain services, which otherwise should have been offered free of charge in the public sector [3]. The requests for informal payments can be initiated by doctors, such as when they consider that their salaries are low [4] or by patients who consider that they will receive better care [5]. This is not some minor activity rarely occurring. In 2020 in the European Union the highest rate of informal payments, used in the form of bribes, was encountered in the medical sector (6% of all respondents) [6], with 22% of patients in Romania making such payments and 19% in Bulgaria [6].

Previous studies have analysed the socio-demographic characteristics of patients making informal payments [7,8,9,10,11,12] and identified a wide array of factors that determine the prevalence of informal payments in different institutional environments. These studies cover both formal institutions (e.g., reduced monitoring of activities in the health sector [13]; lack of efficient management [13]; low level of legal penalties applied to people who have an illegal behaviour [13]; state regulations, which lead to excessive bureaucracy [14]) and informal institutions (e.g., personal factors, such as the desire to do something that is against the law or the desire to get rid of a problem, patients’ perceptions, beliefs, attitude, feelings and relationships) [14,15] or even the lack of alignment between the formal and informal institutions [16]. However, no previous study has so far analysed the factors that influence the prevalence of informal payments in healthcare during a major disruptive event such as a global pandemic.

To fill this gap, the aim of this paper is to evaluate for the first time the drivers of informal payments during the COVID-19 pandemic. To do this, an institutional theory framework is used which investigates the role of formal and informal institutions (focusing on three main issues: trust, transparency and performance) to better understand the determinants of informal payments in the healthcare sector and evaluate the policy initiatives required for reducing informal payments made by the users of public clinics or hospitals.

To evaluate the role of the institutional environment in the prevalence of informal payments in healthcare, the next section briefly describe the previous literature on informal payments made by patients for healthcare services and consequently, the hypotheses to be tested. This is followed by an explanation of the materials and methods used for testing these hypotheses. The third section then reports the results of both the descriptive statistics and multivariate analysis. The final section discusses the findings and draws conclusions of the implications of these findings.

### 1.1. Acceptability of Corruption

Starting with the role of informal institutions, previous studies show that informal payments can be considered a means by which patients hope to receive better care [5]. At the same time, some patients consider that they can benefit from care faster if they pay an additional fee, in the form of an informal payment, while others believe that if they do not make such a payment, they face the risk of not receiving the needed treatment [17]. Similarly, motivated by the low salaries received by the medical staff, patients can offer these informal payments as recognition of the efforts they make, even in the form of a gift [17]. But why should these additional non-taxable payments occur for a medical act that is free in the public sector? One of the most important factors that influences people’s behavior and, implicitly, the decisions they make, is their culture. It is represented by the norms, values and beliefs that people have. This guides them to make certain choices in life, about what is good or bad, and about what is acceptable and what is not [18]. As such, corrupt practices and behavior are deemed acceptable in some societies but not in others. Indeed, gifts or the usage of personal connections to get preferential access to public resources are seen as ‘natural’ in some post-Soviet spaces [19,20]. Similarly, decisions related to informal payments, can be made and deemed acceptable by some populations or by some cultures. This is explained by the fact that social norms inform individuals about the behavior of their peer citizens. Previous findings show that if an individual perceives that others do not behave in a compliant manner, their acceptability of the respective noncompliant behavior will be altered and the likelihood of engaging in similar behavior will increase [21,22,23]. Indeed, a previous extensive literature review displays that features such as patients’ perceptions, beliefs, attitude, feelings, willingness and relationships have been found to be related to the prevalence of informal payments in healthcare [15]. As such we propose to test the following hypothesis:

**Hypothesis** **1** **(H1).** *During a pandemic, informal payments are more likely to occur with higher acceptability of corruption.*

### 1.2. Trust in Public Authorities

Another important driver of informal payments identified in previous studies is represented by lost trust or a low level of trust in public authorities [15]. In general, it is considered that there is an indirect link between trust and corruption [24]. Thus, if the trust in the authorities is lower, the level of corruption in a country is higher. However, other studies conclude that some issues, such as low trust, can be at the same time causes as well as consequences of corruption [25]. As such, a low level of trust can lead to corrupt behaviors but, at the same time, the perception of a high level of corruption further deteriorates an individual’s trust, resulting in a vicious circle. Indeed, previous studies show that the lack of trust in formal institutions alters individuals’ view on social norms and thus, encourages dishonest behaviors. More than that, it has been found that social trust is shaped over time by trust in formal institutions [26]. According to a report in 2020, the year the COVID-19 pandemic broke out, only 48% of EU citizens trusted their national authorities [27]. Among the factors that could determine this level of confidence are socio-demographic characteristics, such as age or level of education [28]. At the same time, taking into account the pandemic context, trust in the authorities was influenced by the following factors: ‘access to information’; ‘socio-economic resilience’; ‘pandemic response measures and enforcement systems’; ‘governmental service provision’ [29]. Thus, the political measures taken by governments regarding the COVID-19 pandemic [30,31], and the attitude of those in citizens’ social networks are also correlated with the degree of trust in public authorities [30]. As such, we propose the following hypothesis:

**Hypothesis** **2** **(H2).** *During a pandemic, informal payments are more likely to occur when patients display low trust in authorities.*

### 1.3. Perceived Transparency

As in the case of trust in the authorities, transparency and accountability are also determinants of informal payments [15]. This type of payment is more common when there is a lack of institutional transparency and accountability [27,32]. Therefore, we can say that there is an inverse link between institutional transparency in a given state and informal payments. Indeed, the risk of corruption is diminished if more information about the decisions taken by public actors is visible and available to the general public [33]. In the European Union, during the COVID-19 pandemic, only four out of ten people categorized the activity of their national governments as transparent. The worst scores were recorded in countries such as France, Poland or Spain [27]. However, even if transparency is an indispensable factor in the medical system, it is sometimes not enough to diminish corruption and create accountability among related people [34]. As such, we propose the following hypothesis:

**Hypothesis** **3** **(H3).** *During a pandemic, informal payments are more likely to occur when the transparency of how the pandemic is handled is perceived as low by the patients.*

### 1.4. Health System Performance

Turning to the role of formal institutions, another important determinant of informal payments has been found to be the performance of the health system, including issues such as quality and access to healthcare services, poor human resource management, lack of medicine and other medical supplies, and an inefficient patient complaint process [15]. Informal payments can be considered as potentially triggered by these problems created in the health system [5]. Usually, performance is characterized by the way in which medical systems are governed [35]. When informal payments are used, the medical system is considered to be degraded, requiring high costs [36]. One factor that could improve the performance of the health system is transparency, the determinant previously characterized [34]. Also, the performance of a health system can be directly correlated with patient satisfaction [37]. When their satisfaction of the services they benefited from is high, the performance will be considered higher, and the probability of informal payments being made by patients is lower. However, when resources are lacking, consumer satisfaction will be limited, as the quality of services provided will be low in this case [38]. Thus, patients will be determined to pay additional amounts to benefit from higher quality services [39]. Based on these finding we propose the following hypothesis:

**Hypothesis** **4** **(H4).** *During a pandemic, informal payments are more likely to occur when the health system performance is poor.*

**Hypothesis** **4a** **(H4a).** *Informal payments are more likely to occur when the access and quality of public healthcare services are poorer.*

**Hypothesis** **4b** **(H4b).** *Informal payments are more likely to occur when the pandemic mortality rate is higher.*

## 2. Materials and Methods

This paper uses the results of The Global Corruption Barometer (GCB)—European Union 2021, involving 40,663 respondents in 27 Member States of the European Union (EU-27). Out of these, 25,744 respondents used the services of a public clinic or hospital in the past 12 months before the survey took place. We confine the analysis here to the respondents from Central and Eastern Europe (18,012 respondents out of which 10,859 were patients). The survey focused on corruption practices and was conducted during the COVID-19 pandemic (from mid-October to December 2020) on behalf of Transparency International. The sample includes respondents aged 18 years old or older and it is representative at a regional level, including a minimum of 300 respondents at NUTS 1 level (Eurostat’s Nomenclature of Territorial Units for Statistics classification). This sampling design ensured that the sample obtained mirrors the population parameters in relation to age, gender, working status and educational level (for details, [40]).

A logistic regression analysis has been employed for analysing the link between informal payments and the formal and informal institutions. The dependent variable is a dichotomous variable displaying whether or not the respondent made informal payments to get assistance or services needed from a public clinic or hospital in the past 12 months prior to the survey. As such, considering that by mid-March 2020, Europe was at the centre of the pandemic, classified as an international emergency by the World Health Organization even from January 2020, it is reasonable to assume that most of the cases where public clinics and hospitals were used in the past 12 months prior to survey by the respondents fall under the COVID-19 pandemic period [41]. Moreover, the institution collecting the data used in this paper acknowledges the fact that contact patterns with public healthcare services are affected by the lockdown measures during the COVID-19 pandemic [27].

The main independent variables for testing the proposed hypotheses are:

*Informal institutions*

- Acceptability of Corruption Index—measures the acceptability of corrupt behaviour and records the answers on how ‘acceptable is it for the government to engage in corruption as long as it delivers good results’ (for testing Hypothesis 1) [6];
- Trust Index—a constructed index measuring the trust in public authorities based on patients self-assessed trust level in the national and local government (for testing Hypothesis 2) [6]; and
- Perceived Transparency Index—measures the perceived transparency by patients on how the government handled the COVID-19 pandemic (for testing Hypothesis 3) [6].

*Formal institutions*

- Access and Quality Index—macro-level index constructed by two indicators: (a) access or the extent to which medical services can be accessed by those who need them, and (b) the quality in delivery healthcare services (for testing Hypothesis 4a) [42]; and
- COVID-19 Mortality Index—macro-level index measuring the mortality rate due COVID-19 deaths per 100,000 inhabitants (for testing Hypothesis 4b) [43].

All indices were normalized on a scale from 0 to 1, where 1 means the highest positive outcome (i.e., lowest acceptability of corruption, highest trust in public institutions, highest perceived transparency on how the government handled the COVID-19 pandemic, easiest access and high quality of public healthcare services, and lowest mortality rate due to COVID-19) and 0 means the lowest negative outcome.

Based on previous literature on informal payments in the healthcare sector [44,45,46,47,48], the other independent variables used as controls include: gender, age, education, employment status, household income, and area of residence. Details about these variables are available in Table A1.

The analysis involved an additive fashion approach for adding the socio-demographic characteristics of the respondents and the indices measuring the formal and informal institutions for assessing their effect on the prevalence of the informal payments. In addition, to better display the relationship between the prevalence of informal payments made for services of a public clinic or hospital and the formal and informal institutions, predicted probabilities of making informal payments in accordance with the institutional setting were graphically displayed. Below, we report the results of the analysis.

## 3. Results

### 3.1. Descriptive Analysis

Table 1 provides an overview of the informal payments made by the users of public clinics or hospitals in the EU member states. Out of 40,663 respondents participating in the survey, 25,744 accessed the services of a public clinic or hospital in the past 12 months prior to the survey. The practice is found to be more prevalent in East-Central Europe (12% of those accessing the services of a public clinic or hospital made informal payments) and less prevalent in Nordic nations (1%). These results are not surprising in the light of previous findings that reveal the prevalence of this practice is higher in East European countries and in post-communist societies [19,49,50]. However, these results should be treated as lower bound estimates considering that the investigated issue is illegal and some of the respondents might be reluctant in openly admitting their involvement in such a practice [51]. Interesting, however, is that when looking to how often this practice was employed by those declaring making informal payments, in Nordic nations the patients making informal payments employ this behavior on a more regular basis (20% of those declaring making such payments) compared with other EU 226 regions (18% in Southern Europe, 15% in East-Central Europe and 11% in Western Europe). Nevertheless, this needs to be cautiously interpreted considering the low prevalence of the practice in the Nordic nations.

Turning to the cross-country variation, Table 2 reveals high differences, from 2% making informal payments of those who accessed the services of a public clinic or hospital in Estonia to 19% in Bulgaria, Hungary and Lithuania and 22% in Romania. Variations also exist in terms of how often these informal payments are made for services of a public clinic or hospital services. This practice proves to be rather an exception, being made only once or twice in Slovenia (84% of those making informal payments). Meanwhile, informal payments represent rather a norm being made a few times or even often by those declaring making such informal payments for a public clinic or hospital among patients in Romania (63%), Latvia (52%), Bulgaria (48%) and Croatia (44%).

Table 3 starts to investigate the link between the prevalence of the informal payments for the services of a public clinic or hospital and the formal and informal institutions. Starting with the informal institutions, the finding is that those not making informal payments have a higher Acceptability of Corruption Index (i.e., a lower acceptability of corrupt behaviour). Those not making informal payments have a higher Trust Index (0.47) compared with those making informal payments (0.38). Also, those not making informal payments perceived a higher transparency on how the government handled the COVID-19 pandemic compared with those making such informal payments (Transparency Index of 0.50 compared with 0.37). This is similarly the case when moving to the assessment of the healthcare system performance and starting to analyse formal institutions. The informal payments are less prevalent in those healthcare systems with easy access and high quality of healthcare services as well as a lower mortality rate caused by the COVID-19 pandemic. As such, the tentative finding is that formal and informal institutions are directly linked to the propensity of making informal payments for services of a public clinic or hospital.

### 3.2. Multivariate Analysis

In order to investigate whether the tentative descriptive results hold when other variables are taken into account and kept constant, Table 4 provides the results of a logistic regression analysis. For testing the reliability of the results, the analysis follows an additive fashion by gradually adding the socio-demographic characteristics of the respondents and the informal institutions, followed by adding in turn the formal institutions. Starting with the influence of the socio-demographic characteristics and informal institutions, Model 1 shows that older age groups are less likely to make informal payments. Meanwhile, those facing financial difficulties, those with a higher education and those living in a small or middle-sized town are more likely to make such payments. No significant differences were identified in relation to gender and employment status. Turning to the institutions, and starting with the informal institutions, the finding is that those having a higher Acceptability of Corruption Index (i.e., a low acceptability of corrupt behaviour) are less likely to make informal payments for services of a public clinic or hospital, validating Hypothesis 1. Models 2 to 5 add, in turn, the other indicators measuring the informal institutions as well as measuring the formal institutions alongside the socio-demographic characteristics and the Acceptability of Corruption Index. The results for the socio-demographic characteristics remain broadly the same and the Acceptability of Corruption Index remains unchanged, showing the robustness of the findings. Starting with Model 2, Hypothesis 2 is validated. Those with higher trust in public authorities are less likely to make informal payments for services of a public clinic or hospital. Similarly, as Model 3 displays, those who perceived a higher transparency on how the government handled the COVID-19 pandemic are less likely to make informal payments for services of a public clinic or hospital (validating Hypothesis 3). Moving to the country-level indicators measuring the health system performance (i.e., formal institutions), as Model 4 and Model 5 display, the results are in the same direction as the descriptive analysis indicated. The individuals are less likely to make informal payments for the services of a public clinic or hospital with healthcare systems which provide easy access and high quality of healthcare services (validating Hypothesis 4a) as well as lower mortality rate caused by the COVID-19 pandemic (validating Hypothesis 4b).

In order to further demonstrate the validity and the robustness of the findings, Table 5 provides the results of various alternative regression methods. The first column (the logistic regression applied on weighted data) represents the approach employed in this paper and is detailed in Table 4. However, if an alternative method is applied, such as using the logistic regression without weighting the data (with or without clustering the data by country) or using it with imputed data where missing, similar results are obtained. Similarly, if employing a probit regression (with or without weighting scheme) taking into account the issue of a potential sample selection (i.e., the fact that only the subsample of individuals who used the services of a public clinic or hospital has been analysed), the results on the significance and the direction of the influence of formal and informal institutions on the prevalence of informal payments made by patients remain unchanged. The results of testing the proposed hypotheses are summarised in Table 6.

**Figure 1:**
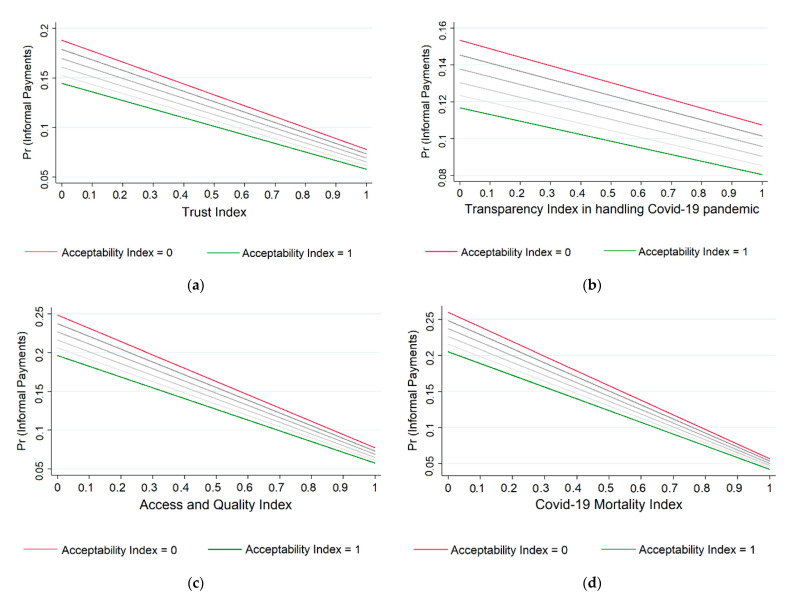
Predicted probability to make informal payments by a ‘representative’ patient in Central and Eastern Europe, by Acceptability of Corruption Index and: (**a**) Trust Index (trust in authorities); (**b**) Transparency Index (transparency in handling COVID-19 pandemic); (**c**) Access and Quality Index (health system performance: access and quality); (**d**) COVID-19 Mortality Index (health system performance: COVID-19 deaths/100,000 inhabitants). Notes: After logistic regression. The ‘representative’ patient in Central and Eastern Europe was obtained by taking the mean and mode of the socio-demographic characteristics as displayed in Table A1, namely: female, 49 years old, primary or secondary educated, working full time, having enough money to buy what is needed and living in a large town. Source: author’s calculations using data from [6,42,43].

To further display the influence of the formal and informal institutions on the prevalence of informal payments made by patients, Figure 1 displays the predicted probability to make informal payments by a ‘representative’ patient in Central and Eastern Europe by informal institutions and formal institutions (trust in authorities; transparency in handling the COVID-19 pandemic; health system performance: access and quality and COVID-19 deaths/100,000 inhabitants). The results show that the probability of a patient making informal payments decreases with better informal institutions (i.e., less acceptability of corrupted behaviour) and better formal institutions (i.e., higher trust in authorities, higher perceived transparency in handling the COVID-19 pandemic, easier access and higher quality of healthcare services and lower mortality rate due to the COVID-19 pandemic).

## 4. Discussion

This paper has highlighted the prevalence of informal payments during the COVID-19 pandemic. Due to the specificity of the period, committing acts of corruption can be an opportunity for those who are accustomed to this practice [52]. Indeed, the finding of this paper is that 12% of the patients in Central and Eastern Europe made at least one informal payment during the analysed period. As the authorities’ efforts are more focused on taking measures to prevent new coronavirus infections, acts of corruption such as informal payments can be considered an ignored pandemic [53,54]. This emphasizes the need to apply measures to discourage the use of informal payments. To achieve the goal of reducing the use of informal payments, these measures should be tailored by the type of determinants that generate their occurrence.

Firstly, the way in which informal payments are perceived by citizens should be changed, especially among those with financial difficulties or better education. Older people will have to be excluded from this category, as they are less likely to make such payments. In general, patients are tempted to follow the provisions of unwritten rules, being supported by the institutional asymmetry of the country in which they live [55,56]. In this regard, various steps can be taken to change the behavioural intentions of consumers and reduce institutional asymmetry, thus improving personal norms, such as: information campaigns for vulnerable people about the costs and adverse events that may occur when informal payments are used, or by making normative appeals to the parties involved to diminish their intentions to use such practices [56].

Secondly, building trust in public authorities is needed. This needs a major effort by the authorities considering that, during this period, the degree of trust in the authorities is directly proportional to the way in which citizens perceive the management of the COVID-19 pandemic [57]. Furthermore, people who are not in good health are reluctant to trust the government [28]. Given that the main threat of the COVID-19 pandemic is to endanger human health, the authorities’ effort will be twofold. Thus, if the authorities will be able to manage the pandemic issue in an efficient manner, the risk of infection will be lower, and consequently, the trust of the citizens will be higher. As documented by previous studies, some measures that could foster trust include accurate monitoring of the delivery process, enforcing precise control mechanisms and promoting accountability in the health system [15].

Also, another measure to reduce the spread of informal payments is to increase transparency. During the pandemic, this was a delicate subject. The most important doubts were those related to the distribution of the vaccine and those related to the development of vaccination campaigns, which could have given rise to certain risks of corruption [58]. Through an increase in transparency in the health sector, patients will be able to make a comparison between providers in the market, to make the most appropriate and effective choices [59]. However, there may be some limitations. Transparency is based on public reporting by the authorities, and to be effective, it should play the following roles: providing information that can be easily understood; published data should be clearly targeted to those who need them, not to the whole mass of patients; the existence of financial interventions by political decision-makers [60]. Civil society organisations can take a direct role in improving trust in the way that authorities manage the COVID-19 pandemic and increasing transparency through acting as ‘watch-dogs’ by monitoring the public procurement contracts as well as the supply chain of the medicines and supplies needed [61].

Finally, the improvement of the performance in the health system must be considered if informal payments in healthcare are to be tackled. Often, this is translated into the quality of the services provided by doctors. One solution could be to collect formal fees from patients, and to reinvest these funds to improve medical facilities [39]. In this way, the prevalence of informal payments would be reduced, and patients would have the chance to benefit from better services. Additional funding may also come from the authorities themselves. They could support health systems by providing financial incentives or even by adopting initiatives, which can take different forms: ‘regulation and self-regulation’; ‘guidelines’; ‘performance targets’ [62]. At the same time, taking into account another factor mentioned above, improving the performance of the health system can also lead to a strengthening of trust in the authorities. These things can happen through more in-depth knowledge of the experiences, needs and aspirations that citizens have [63].

The analysis in this paper, however, has been unable to explore the cultural issues that affect the decision of making informal payments for the services of a public clinic or hospital. Future qualitative research (using for example, ethnography) is necessary for complementing these quantitative findings by exploring in depth the cultural issues and social norms that influence the decision to make informal payments for healthcare services.

## 5. Conclusions

As a result of its characteristics, the COVID-19 pandemic has proven to be a productive period for committing acts of corruption, especially in the use of informal payments. This paper has advanced the understanding of informal payments by employing the institutional theoretical framework in a particular period, when the global healthcare systems have been disrupted by the COVID-19 pandemic. The findings show that the high acceptability of corruption, lower trust in authorities, lower perceived transparency in handling the COVID-19 pandemic (i.e., informal institutions), as well as difficult access and poor quality of healthcare services, and higher mortality rate due to the COVID-19 pandemic (i.e., formal institutions) are directly linked to a higher prevalence of informal payments in healthcare. However, a potent future research avenue is represented by an analysis of the interdependences between the various determinants of the informal payments identified in this paper.

Whether the results of this paper are valid when evaluating other regions beyond Central and Eastern Europe needs to be evaluated. If this paper encourages scholars to employ the institutional theory framework in other geographical areas, then one of the intentions of this paper has been achieved. If the paper also encourages the policy makers to tailor their policy measures for tackling the informal payments in accordance with the current state of the institutional environment they operate in, then the paper will have fulfilled its wider intention.

## Figures and Tables

**Table 1:** Public clinic or hospital users making informal payments, by EU region (*n* = 25,744; %).

Region	Informal Payments	Informal Payments:		
		Once or Twice	A Few Times	Often
Central and Eastern Europe	12	56	29	15
Nordic Nations	1	57	23	20
Southern Europe	4	55	27	18
Western Europe	3	66	23	11
EU-27	6	57	28	15

Source: author’s calculations using data from [6].

**Table 2:** Public clinic or hospital users making informal payments, by Central and Eastern European countries (*n* = 10,859; %).

Country	Informal Payments	Informal Payments:		
		Once or Twice	A Few Times	Often
Romania	22	37	37	26
Bulgaria	19	52	34	14
Hungary	19	58	19	23
Lithuania	19	57	37	6
Croatia	15	56	20	24
Czechia	10	69	26	5
Latvia	10	48	33	19
Poland	10	59	34	7
Slovakia	10	66	28	6
Slovenia	5	84	12	4
Estonia	2	65	24	11
Central and Eastern Europe	12	56	29	15

Source: author’s calculations using data from [6].

**Table 3:** Informal payments and institutions (*n* = 10,859; indexes).

Index	Informal Payments	
	Yes	No
Acceptability of Corruption Index	0.67	0.72
Trust Index (Trust in public authorities)	0.38	0.47
Transparency Index (In handling COVID-19 pandemic)	0.37	0.50
*Health system performance*		
Access and Quality Index (For public healthcare services)	0.52	0.54
COVID-19 Mortality Index (COVID-19 deaths/100,000 inhabitants)	0.42	0.48

Notes: normalized indexes 0 to 1, where 1 = highest positive outcome. Source: author’s calculations using data from [6,42,43].

**Table 4:** Logistic regression of the probability to make informal payments for public healthcare services in Central and Eastern Europe.

	Model 1					Model 2				
Fixed Part	Coef.		SE	OR	(OR, 95% CI)	Coef.		SE	OR	(OR, 95% CI)
*Socio-demographic control variables*										
Gender (R: Male)										
Female	0.022		0.082	1.022	(0.870–1.200)	0.034		0.083	1.035	(0.880–1.217)
Age	−0.011	***	0.004	0.989	(0.982–0.996)	−0.009	**	0.004	0.991	(0.984–0.998)
Education (R: Primary, Secondary)										
Tertiary	0.207	**	0.087	1.230	(1.037–1.458)	0.251	***	0.088	1.285	(1.082–1.527)
Employments status (R: Working full-time)										
Working part-time	0.129		0.188	1.138	(0.787–1.647)	0.170		0.189	1.185	(0.819–1.716)
Not working (seeking)	−0.181		0.212	0.835	(0.551–1.264)	−0.179		0.211	0.836	(0.553–1.265)
Retired	−0.011		0.137	0.989	(0.756–1.294)	−0.023		0.138	0.978	(0.746–1.281)
Not working (not seeking)	−0.210		0.317	0.811	(0.436–1.508)	−0.270		0.327	0.764	(0.402–1.449)
Student	−0.125		0.232	0.883	(0.561–1.390)	−0.037		0.232	0.964	(0.611–1.520)
Homemaker	−0.094		0.201	0.910	(0.614–1.349)	−0.060		0.202	0.942	(0.633–1.400)
Household income (R: Enough to buy what wanted)										
Enough to buy what needed	0.021		0.106	1.021	(0.830–1.257)	−0.034		0.106	0.967	(0.785–1.190)
Facing difficulties	0.420	***	0.113	1.521	(1.218–1.900)	0.256	**	0.116	1.291	(1.029–1.621)
Area (R: Rural area or village)										
Small, middle-sized town	0.268	**	0.105	1.307	(1.064–1.606)	0.237	**	0.106	1.267	(1.030–1.560)
Large town	0.186	*	0.103	1.205	(0.985–1.474)	0.165		0.104	1.179	(0.962–1.445)
*Tested Hypotheses*										
Acceptability Index ^1^	−0.270	***	0.090	0.764	(0.641–0.910)	−0.247	***	0.091	0.782	(0.654–0.934)
Trust Index ^2^						−1.492	***	0.174	0.225	(0.160–0.316)
Constant	−1.605	***	0.201			−1.010	***	0.210		
Observations	10,109					10,070				
F	4.86					9.39				
Prob. > F	0.000					0.000				
	**Model 3**					**Model 4**				
**Fixed Part**	**Coef.**		**SE**	**OR**	**(OR, 95% CI)**	**Coef.**		**SE**	**OR**	**(OR, 95% CI)**
*Socio-demographic control variables*										
Gender (R: Male)										
Female	0.019		0.083	1.019	(0.865–1.200)	0.008		0.084	1.009	(0.856–1.190)
Age	−0.008	**	0.004	0.992	(0.985–0.999)	−0.007	**	0.004	0.993	(0.986–1.000)
Education (R: Primary, Secondary)										
Tertiary	0.237	***	0.089	1.267	(1.064–1.509)	0.276	***	0.090	1.318	(1.105–1.571)
Employments status (R: Working full-time)										
Working part-time	0.136		0.191	1.146	(0.788–1.666)	0.183		0.194	1.201	(0.820–1.758)
Not working (seeking)	−0.116		0.211	0.890	(0.589–1.346)	−0.133		0.212	0.876	(0.577–1.328)
Retired	−0.022		0.140	0.978	(0.744–1.287)	−0.060		0.140	0.941	(0.715–1.239)
Not working (not seeking)	−0.352		0.340	0.704	(0.362–1.369)	−0.315		0.339	0.729	(0.375–1.419)
Student	−0.041		0.231	0.960	(0.611–1.508)	0.018		0.228	1.018	(0.651–1.593)
Homemaker	−0.124		0.211	0.883	(0.584–1.336)	−0.138		0.209	0.871	(0.579–1.312)
Household income (R: Enough to buy what wanted)										
Enough to buy what needed	−0.047		0.107	0.954	(0.773–1.177)	−0.034		0.108	0.966	(0.782–1.193)
Facing difficulties	0.267	**	0.117	1.306	(1.039–1.641)	0.209	*	0.118	1.233	(0.979–1.553)
Area (R: Rural area or village)										
Small, middle-sized town	0.204	*	0.107	1.226	(0.994–1.513)	0.121		0.110	1.129	(0.910–1.401)
Large town	0.138		0.105	1.148	(0.934–1.410)	0.066		0.107	1.068	(0.866–1.317)
*Tested Hypotheses*										
Acceptability Index ^1^	−0.284	***	0.093	0.752	(0.628–0.902)	−0.323	***	0.093	0.724	(0.603–0.869)
Trust Index ^2^	−1.235	***	0.195	0.291	(0.198–0.426)	−1.027	***	0.196	0.358	(0.244–0.526)
Transparency Index ^3^	−0.403	***	0.104	0.668	(0.545–0.819)	−0.419	***	0.106	0.657	(0.534–0.809)
Health system Performance										
Access and Quality Index ^4^						−1.437	***	0.195	0.238	(0.162–0.349)
COVID-19 Mortality Index ^5^						−1.823	***	0.211	0.161	(0.107–0.244)
Constant	−0.923	***	0.213			0.637	**	0.279		
Observations	9794					9794				
F	9.34					14.66				
Prob. > F	0.000					0.000				

Notes: Significant at * *p* < 0.1, ** *p* < 0.05, *** *p* < 0.01; Coefficients compared to the reference category (R) shown in brackets; ^1^ Acceptability of Corruption Index; ^2^ Trust in public authorities—Trust Index; ^3^ Transparency in handling COVID-19 pandemic—Transparency Index; ^4^ Health system performance: Access and Quality; ^5^ COVID-19 deaths/100,000 inhabitants—Mortality Index. Source: author’s calculations using data from [6,42,43].

**Table 5:** Sensitivity analysis.

	Logistic Regression				Probit Regression with Sample Selection	
	With Weighting Scheme	Without Weighting Scheme	Without Weighting Scheme	Imputed Missing Data	With Weighting Scheme	Without Weighting Scheme
Socio-demographic control variables	Yes	Yes	Yes	Yes	Yes	Yes
Tested Hypotheses						
Acceptability Index ^1^	−0.323 *** (0.093)	−0.451 *** (0.068)	−0.451 *** (0.059)	−0.319 *** (0.093)	−0.177 *** (0.050)	−0.250 *** (0.038)
Trust Index ^2^	−1.027 *** (0.196)	−1.143 *** (0.140)	−1.143 *** (0.105)	−1.034 *** (0.187)	−0.557 *** (0.105)	−0.622 *** (0.076)
Transparency Index (COVID-19) ^3^	−0.419 *** (0.106)	−0.192 *** (0.073)	−0.192 ** (0.080)	−0.392 *** (0.102)	−0.219 *** (0.056)	−0.103 *** (0.039)
Health System Performance						
Access and Quality Index ^4^	−1.437 *** (0.195)	−1.555 *** (0.162)	−1.555 *** (0.499)	−1.390 *** (0.191)	−0.789 *** (0.108)	−0.855 *** (0.089)
COVID-19 Mortality Index ^5^	−1.823 *** (0.211)	−1.551 *** (0.158)	−1.551 ** (0.619)	−1.736 *** (0.201)	−0.997 *** (0.113)	−0.868 *** (0.087)
Clustered by country (11)			Yes			
Selection equation ^6^					Yes	Yes
Observations	9794	9794	9794	10,859	16,866	16,866
Censored						7072
Uncensored						9794
Imputations (multivariate)				Yes		
Prob. > F/chi2	0.000	0.000	0.000	0.000	0.000	0.000

Notes: Significant at ** *p* < 0.05, *** *p* < 0.01; Coefficients compared to the reference category (R) in brackets; Standard errors displayed in parentheses; ^1^ Acceptability of Corruption Index; ^2^ Trust in authorities—Trust Index; ^3^ Transparency in handling COVID-19 pandemic—Transparency Index; ^4^ Health system performance: access and quality; ^5^ Health system performance: COVID-19 deaths/100,000 inhabitants—Mortality Index; ^6^ Socio-demographic variables included in the selection equation: age, education. Source: author’s calculations using data from [6,42,43].

**Table 6:** Summary of the results.

Hypothesis	Result
H1: During a pandemic, informal payments are more likely to occur with higher acceptability of corruption.	Confirmed
H2: During a pandemic, informal payments are more likely to occur when patients display low trust in authorities.	Confirmed
H3: During a pandemic, informal payments are more likely to occur when the transparency of how the pandemic is handled is perceived as low by the patients	Confirmed
H4: During a pandemic, informal payments are more likely to occur when the health system performance is poor.	
H4a: Informal payments are more likely to occur when the access and quality of public healthcare services are poorer.	Confirmed
H4b: Informal payments are more likely to occur when the pandemic mortality rate is higher.	Confirmed

## Data Availability

Publicly available datasets were analysed in this study. This data can be found here: https://www.transparency.org/en/gcb/eu/european-union-2021/press-and-downloads, accessed on 1 August 2021.

## Appendix A

**Table A1:** Analysed variables (*n* = 10,859).

Variable	Variable Type	Mode or Mean	Min, Max
*Socio-demographic control variables*			
Gender	Dummy	Female (56%)	0, 1
Age	Numeric	49 years	18, 105
Education	Dummy	Primary, Secondary (67%)	0, 1
Employment status	Categorical	Working full-time (52%)	1, 7
Household income	Categorical	Having enough to buy what needed (45%)	1, 3
Area	Categorical	Large town (36%)	1, 3
*Index*			
Asymmetry Index	Normalized Index 0 to 1 (0 = high asymmetry level—formal-informal institutions; 1 = low asymmetry level − formal-informal institutions).	0.72	0, 1
Trust Index	Normalized Index 0 to 1 (0 = low level of trust in public authorities (local and national government); 1 = high level of trust in public authorities (local and national government)).	0.46	0, 1
Transparency Index	Normalized Index 0 to 1 (0 = low level of transparency in handling COVID-19 pandemic; 1 = high level of transparency in handling COVID-19 pandemic).	0.49	0, 1
Access and Quality Index	Normalized Index 0 to 1 (0 = low health system performance in terms of access to and quality of public healthcare services; 1 = high health system performance in terms of access to and quality of public healthcare services). Access = extent to which medical services can be accessed by those who need them. Quality = quality in delivery healthcare services.	0.54	0, 1
COVID-19 Mortality Index	Normalized Index 0 to 1 (0 = high COVID-19 mortality rate (COVID-19 deaths/100,000 inhabitants); 1 = low COVID-19 mortality rate (COVID-19 deaths/100,000 inhabitants).	0.48	0, 1
